# Creatinine Decline Rate as a Predictor of Renal Recovery After Acute Kidney Injury: A Retrospective Cohort Study

**DOI:** 10.3390/diagnostics15222904

**Published:** 2025-11-17

**Authors:** Kürşad Öneç, Gülşah Altun, Tansu Sav

**Affiliations:** Department of Nephrology, Faculty of Medicine, Duzce University , Duzce 81620, Türkiye; gulsahaltuntas11@gmail.com (G.A.); savtansu@gmail.com (T.S.)

**Keywords:** acute kidney injury, creatinine decline rate, renal recovery, chronic kidney disease, biomarker, prognosis

## Abstract

**Background/Objectives**: Acute kidney injury (AKI) is a common complication in hospitalized patients and carries a substantial risk of chronic kidney disease (CKD), dialysis dependence, and mortality. Although novel biomarkers such as NGAL, KIM-1, and cystatin C have shown promise, their high cost and limited availability restrict their use in routine practice, particularly in developing countries where CKD incidence is rising. The trajectory of serum creatinine decline after its peak may provide a simple, low-cost, and universally available prognostic marker for renal recovery. **Methods**: This retrospective cohort study included 817 adult patients diagnosed with AKI between January 2015 and December 2024. The creatinine decline rate was calculated as the difference between peak and discharge creatinine divided by hospital stay (mg/dL/day). Patients were stratified into rapid or slow decline groups according to the median value (0.19 mg/dL/day). Post-discharge outcomes, including CKD development, readmission, dialysis requirement, and mortality, were evaluated at 3, 6, and 12 months. Receiver operating characteristic (ROC) analysis was performed to determine the optimal cutoff for predicting renal recovery. **Results**: Patients in the rapid decline group (*n* = 409) were younger and had fewer comorbidities and shorter hospital stays than those in the slow decline group (*n* = 408). The ROC analysis yielded an AUC of 0.78 (95% CI 0.73–0.82, *p* < 0.001) with an optimal cutoff of 0.18 mg/dL/day (sensitivity 76%, specificity 71%). At 12 months, CKD (18.6% vs. 34.3%), dialysis requirement (3.4% vs. 8.8%), readmission (29.8% vs. 41.2%), and mortality (9.3% vs. 14.2%) were all significantly higher in the slow decline group (all *p* < 0.05). In multivariable analysis, faster creatinine decline independently predicted renal recovery (OR = 1.36 per 0.1 mg/dL/day, 95% CI 1.22–1.53, *p* < 0.001), along with younger age, higher serum albumin, and shorter hospital stay. In the longitudinal GEE model, both time (*p* = 0.004) and group effects (*p* < 0.001) remained significant, with an interaction effect (*p* = 0.018) indicating greater eGFR improvement over time among patients with rapid creatinine decline. **Conclusions**: The rate of creatinine decline is an independent predictor of long-term renal recovery following AKI. This simple and inexpensive parameter may complement novel biomarkers and serve as a practical risk-stratification tool in diverse clinical settings, especially where resources are limited. Prospective multicenter studies integrating albuminuria and emerging biomarkers are warranted to validate and expand these findings.

## 1. Introduction

Acute kidney injury (AKI) is a frequent clinical syndrome encountered in hospitalized patients and is associated with significant morbidity, mortality, and progression to chronic kidney disease (CKD) [1,2]. Its incidence has been reported to range from 10% to 20% in hospitalized populations, and it can increase up to 50% in critically ill patients admitted to intensive care units. AKI is not only an acute complication but also an important determinant of long-term renal and patient outcomes, with many survivors at risk of incomplete renal recovery, CKD, and end-stage kidney disease. Early identification of patients who are unlikely to recover optimal renal function remains one of the major challenges in nephrology and critical care [3,4,5].

The prognosis of AKI is highly heterogeneous and depends on several factors, including the severity of renal impairment, underlying comorbidities, hemodynamic status, exposure to nephrotoxic agents, and timely implementation of supportive measures. Traditional markers, such as baseline creatinine levels, peak creatinine, urine output, and need for renal replacement therapy, provide important diagnostic and prognostic information but are often insufficient to accurately predict renal recovery [6,7]. In recent years, both serum and urinary biomarkers such as neutrophil gelatinase-associated lipocalin, kidney injury molecule-1, interleukin-18, and cystatin C have been investigated to improve early prognostication. Although promising, these biomarkers are not routinely available in daily clinical practice due to cost, limited accessibility, and lack of standardization [8,9,10].

Serum creatinine, despite its well-known limitations as a delayed and indirect indicator of kidney function, remains the most widely used and universally available marker in both clinical care and research [11,12]. However, most studies have focused on peak creatinine or baseline-to-peak changes as indicators of AKI severity, whereas the dynamic pattern of creatinine decline during recovery has received comparatively less attention. The rate at which creatinine decreases after its peak may reflect the extent of preserved renal reserve, the capacity for tubular repair, and overall recovery potential. Importantly, a higher absolute or relative peak creatinine value does not necessarily indicate a worse prognosis; in individuals with greater muscle mass or metabolic reserve, it may instead reflect a higher baseline creatinine and preserved renal capacity, particularly when accompanied by faster recovery. In this context, evaluating creatinine decline kinetics could represent a simple, inexpensive, and easily applicable prognostic tool for predicting renal outcomes [12,13,14,15].

A few prior studies have suggested that a slower decline in creatinine during hospitalization may be associated with a higher risk of CKD progression, dialysis dependence, and increased mortality. However, evidence remains limited, and data are often derived from relatively small, heterogeneous cohorts [16,17,18]. Furthermore, there is a lack of standardized definitions for creatinine decline rate, with different studies adopting variable cut-offs and methodologies. This heterogeneity underscores the need for larger, well-characterized cohorts that can provide robust data on the prognostic significance of creatinine decline kinetics in AKI [19,20].

In addition, although creatinine and estimated glomerular filtration rate are key components of kidney function assessment, accurate diagnosis and staging of CKD also require evaluation of albuminuria—ideally via urine albumin-to-creatinine ratio. Future studies combining creatinine kinetics with albuminuria may provide a more comprehensive approach to renal risk stratification.

The present retrospective study aimed to investigate the relationship between in-hospital creatinine decline rate and long-term renal recovery in a large single-center cohort of patients with AKI. Specifically, we evaluated whether the rate of creatinine reduction following its peak could serve as an independent predictor of renal recovery at 12 months, while also assessing its association with important clinical outcomes such as CKD development, hospital readmission, dialysis requirement, and mortality. By focusing on a universally available and inexpensive biomarker, this study sought to provide a practical prognostic measure that could be readily integrated into routine clinical practice.

## 2. Materials and Methods

### 2.1. Study Design, Population, and Ethics

This retrospective cohort enrollment study was conducted at Düzce University Faculty of Medicine Hospital. Although data collection and analysis were retrospective, patients were enrolled based on predefined criteria and evaluated from risk factors to outcomes, consistent with a cohort design. All adult patients (≥18 years) admitted between 1 January 2015 and 31 December 2024 with a diagnosis of acute kidney injury (AKI) were screened.

AKI was defined according to the Kidney Disease: Improving Global Outcomes (KDIGO) 2012 criteria as an increase in serum creatinine by ≥0.3 mg/dL within 48 h, or an increase to ≥1.5 times baseline within 7 days [21].

Patients were eligible if they met the KDIGO criteria for AKI, had at least three separate serum creatinine measurements during hospitalization, and had available follow-up data. Exclusion criteria included chronic hemodialysis or peritoneal dialysis, history of kidney transplantation, pregnancy, or incomplete medical records. The primary underlying etiologies of AKI were also recorded, including sepsis, ischemic, nephrotoxic, postoperative, and multifactorial causes. A total of 817 patients met the inclusion criteria and were included in the final analysis.

The study was carried out in accordance with the ethical principles of the Declaration of Helsinki. Given its retrospective nature, written informed consent was waived. Ethical approval was obtained from the Non-Interventional Clinical Research Ethics Committee of Düzce University (Decision No: 2025/265, Date: 22 September 2025).

### 2.2. Demographic, Clinical, and Laboratory Data

Baseline demographic and clinical data, including age, sex, body mass index (BMI), and comorbidities (hypertension, diabetes mellitus, coronary artery disease, chronic obstructive pulmonary disease, etc.), were collected from electronic medical records.

Laboratory parameters assessed at admission, peak during hospitalization, and at discharge included serum creatinine, blood urea nitrogen (BUN), albumin, hemoglobin, sodium, potassium, and C-reactive protein (CRP). All laboratory tests were performed in the central laboratory using a Roche Cobas^®^ 8000 Modular Analyzer Series (Roche Diagnostics, Mannheim, Germany). Serum creatinine was measured using an enzymatic method, and internal quality controls were routinely performed according to manufacturer guidelines.

The proportion of missing data for each variable was reviewed. Missing values represented <5% for all major laboratory parameters, and no imputation or data replacement was applied. Analyses were conducted using available complete cases.

### 2.3. Assessment of Creatinine Decline Rate

For each patient, serum creatinine values measured during hospitalization were reviewed. The peak creatinine was defined as the highest value recorded during the hospital stay, and the discharge creatinine was the final measurement prior to discharge.

The average daily creatinine decline rate was calculated by subtracting the discharge creatinine from the peak creatinine and dividing the result by the total length of stay, expressed as milligrams per deciliter per day (mg/dL/day). To better capture the overall recovery trajectory, all available creatinine values between the peak and discharge were also analyzed using linear regression, and the resulting slope coefficient was recorded as an alternative measure of daily decline.

In addition, the relative percentage reduction in creatinine was calculated by dividing the difference between peak and discharge creatinine by the peak value and multiplying by 100. The change in renal function over time was further expressed as the difference between discharge and 12-month eGFR (ΔeGFR), providing a continuous measure of renal recovery.

Patients were subsequently stratified into rapid and slow decline groups according to the median daily decline rate (0.19 mg/dL/day).

To further explore the predictive value of this parameter, receiver operating characteristic (ROC) curve analysis was performed, and the Youden index was applied to determine the optimal cut-off point for predicting renal recovery at 12 months [22].

### 2.4. Clinical Follow-Up and Outcomes

Post-discharge outcomes were assessed at 3, 6, and 12 months using outpatient clinic visits, electronic hospital records, and the national e-Nabız health information system.

The primary outcome was long-term renal recovery, defined as an estimated glomerular filtration rate (eGFR) ≥ 60 mL/min/1.73 m^2^ without persistent renal dysfunction.

The secondary outcomes included the development of chronic kidney disease (CKD) according to KDIGO guidelines (eGFR < 60 mL/min/1.73 m^2^ for ≥3 months), hospital readmissions, dialysis requirement, and all-cause mortality. Progression of eGFR as a continuous parameter and ≥5 mL/min/1.73 m^2^ reduction over time were explored descriptively but not included in the primary regression models due to missing intermediate measurements.

### 2.5. Statistical Analysis

All statistical analyses were performed using IBM SPSS Statistics version 25.0 (IBM Corp., Armonk, NY, USA). Continuous variables were tested for normality using the Kolmogorov–Smirnov test and visual methods (histogram and Q–Q plots). Normally distributed variables were expressed as mean ± standard deviation (SD), while non-normally distributed variables were presented as median and interquartile range (IQR). Categorical variables were reported as frequencies and percentages.

Comparisons between the rapid and slow creatinine decline groups were conducted using the independent samples *t*-test for normally distributed continuous variables and the Mann–Whitney U test for non-normally distributed variables. The chi-square test or Fisher’s exact test (where appropriate) was applied for categorical variables.

Repeated measures of eGFR and CKD status across follow-up intervals (3, 6, and 12 months) were analyzed using a Generalized Estimating Equations (GEE) model to account for within-subject correlations and missing-at-random data patterns. The model assessed the main effects of time and group (rapid vs. slow decline) as well as their interaction on renal recovery trajectories. For non-parametric repeated measures, Cochran’s Q test and Friedman’s test were used as appropriate.

Multivariate logistic regression analysis was performed to identify independent predictors of renal recovery at 12 months. Variables with a *p* value < 0.10 in univariate analysis, as well as clinically relevant covariates (age, sex, hypertension, diabetes, albumin, peak creatinine, creatinine decline rate, ICU admission, and length of hospital stay), were included in the regression model. Odds ratios (ORs) with 95% confidence intervals (CIs) were calculated. Model fit was assessed with the Hosmer–Lemeshow goodness-of-fit test, and discriminatory ability was evaluated using the area under the ROC curve.

A two-tailed *p* value < 0.05 was considered statistically significant.

## 3. Results

A total of 817 patients were included in the analysis. As shown in Table 1, patients were stratified into two groups based on the median daily creatinine decline rate (0.19 mg/dL/day): the rapid decline group (*n* = 409) and the slow decline group (*n* = 408). The mean age was significantly lower in the rapid decline group compared to the slow decline group (63.9 ± 12.7 vs. 68.2 ± 13.0 years, *p* < 0.001). The proportion of female patients was also lower in the rapid decline group (41.1% vs. 51.0%, *p* = 0.002). The prevalence of hypertension (52.3% vs. 63.7%, *p* < 0.001) and diabetes mellitus (29.3% vs. 38.7%, *p* = 0.003) was significantly higher in the slow decline group. In addition, intensive care unit (ICU) requirement was more frequent among patients in the slow decline group (33.6% vs. 22.5%, *p* < 0.001), and their median length of hospital stay was longer (10 [7–15] vs. 8 [6–12] days, *p* < 0.001).

The etiological distribution of AKI is presented in Table 2. Sepsis accounted for the largest proportion of AKI cases (32%), followed by ischemic injury (27%), nephrotoxic exposure (19%), postoperative causes (12%), and multifactorial etiologies (10%).

As shown in Table 3, discharge creatinine levels were significantly lower in the rapid decline group (1.4 ± 0.6 vs. 2.0 ± 0.9 mg/dL, *p* < 0.001). The median creatinine decline rate was 0.28 [0.22–0.36] mg/dL/day in the rapid group and 0.12 [0.08–0.15] mg/dL/day in the slow group (*p* < 0.001). Patients with rapid creatinine decline had significantly higher albumin and hemoglobin levels (both *p* < 0.001) and lower urea concentrations (*p* < 0.001).

As shown in Table 4, the cumulative incidence of CKD was higher in the slow decline group at all time points (3, 6, and 12 months, all *p* < 0.001). Similarly, all-cause mortality rates at 3, 6, and 12 months were significantly higher in the slow decline group (*p* = 0.04, *p* = 0.03, and *p* = 0.03, respectively). Hospital readmission and dialysis requirements within 12 months were also more frequent in the slow decline group (*p* < 0.001 and *p* = 0.002, respectively).

Receiver operating characteristic (ROC) curve analysis demonstrated that the creatinine decline rate predicted renal recovery at 12 months with an AUC of 0.78 (95% CI: 0.73–0.82, *p* < 0.001). The optimal cutoff determined by the Youden index was 0.18 mg/dL/day (sensitivity 76%, specificity 71%). Multivariate logistic regression analysis identified faster creatinine decline as an independent predictor of renal recovery (OR = 1.36 per 0.1 mg/dL/day, 95% CI: 1.22–1.53, *p* < 0.001), along with younger age, higher serum albumin, and shorter hospital stay (Table 5).

According to Table 6, the Generalized Estimating Equations (GEE) model revealed that both time (*p* = 0.004) and group (rapid vs. slow decline; *p* < 0.001) effects were statistically significant, while the interaction term (group × time; *p* = 0.018) indicated a greater longitudinal improvement in renal function among patients with rapid creatinine decline. The model used an identity link and exchangeable correlation structure with robust standard errors, adjusting for within-subject correlation and missing-at-random data patterns.

## 4. Discussion

In this retrospective single-center study involving 817 patients with acute kidney injury, we investigated the association between the rate of creatinine decline and long-term renal recovery. Our findings suggest that patients with a more rapid decline in serum creatinine were significantly more likely to achieve renal recovery at 12 months, whereas those with slower declines exhibited higher rates of chronic kidney disease, rehospitalization, and mortality. Multivariable analyses confirmed that the rate of creatinine decline was an independent predictor of long-term renal outcomes. These observations support the notion that the kinetics of creatinine reduction may serve as a clinically relevant marker in the prognostic assessment of AKI, although they must be interpreted with caution.

The literature on creatinine decline kinetics is limited but increasingly recognized [23]. Current evidence indicates that the heterogeneity of renal recovery after AKI is largely determined by the trajectory of creatinine decline, and that slower reductions are associated with an increased risk of long-term loss of kidney function [13,24,25,26,27]. Similarly, Obi et al. emphasized that the slope of creatinine reduction reflects tubular repair capacity and residual renal reserve [28]. Our findings are consistent with these observations and extend them to a larger cohort with longer follow-up. Importantly, the methods used to define creatinine decline vary across studies—some assess the time required for a 50% reduction, others calculate the average daily decline, or use regression slopes. In the present study, both average daily decline and linear regression slope were applied, thereby aligning with previously reported approaches and enhancing robustness [29,30].

Interestingly, our results also highlight that a higher peak or relative rise in serum creatinine should not automatically be interpreted as a marker of poor prognosis. In some patients, particularly those with greater muscle mass or preserved metabolic reserve, a more pronounced initial increase may occur without reflecting irreversible injury. When accompanied by a rapid decline, this pattern could instead indicate an intact renal reserve and effective tubular repair mechanisms. This concept supports the view that creatinine dynamics, rather than isolated values, provide deeper insight into recovery potential.

In addition to creatinine kinetics, other laboratory parameters were also informative. Patients with a faster decline exhibited higher serum albumin and lower CRP and urea levels. The prognostic relevance of hypoalbuminemia has been consistently reported, as it reflects systemic inflammation and malnutrition, both of which compromise renal recovery [31]. Similarly, elevated CRP and urea are established markers of disease severity and were associated with poorer outcomes in our cohort. Taken together, these findings highlight that creatinine decline should not be interpreted in isolation but rather considered in conjunction with systemic clinical and biochemical context [32].

Our findings are consistent with previous reports emphasizing the prognostic importance of renal functional trajectories after AKI. Similarly to earlier studies that demonstrated the association between incomplete recovery or persistent creatinine elevation and increased risk of CKD progression and mortality [13,24,25,26,27,33], the present analysis confirms that the rate of creatinine decline itself carries independent predictive value. Moreover, the magnitude of this association in our cohort (AUC 0.78; OR 1.36 per 0.1 mg/dL/day) aligns closely with prior studies evaluating renal recovery markers [25,26,29]. While most earlier investigations focused on baseline-to-peak creatinine changes or recovery time as prognostic indicators, our study extends this knowledge by demonstrating that post-peak creatinine kinetics—particularly the average daily decline—can provide a simple, low-cost, and clinically meaningful tool for early risk stratification. These results reinforce the concept that renal recovery after AKI should be viewed as a dynamic continuum rather than a binary event, with the speed of biochemical improvement offering valuable insight into long-term outcomes.

From a clinical perspective, long-term outcomes were particularly noteworthy. At 12 months, patients in the slow-decline group showed significantly higher rates of CKD and dialysis dependence, outcomes that align with previous studies linking incomplete renal recovery to increased risk of end-stage kidney disease and mortality [33]. Our study reinforces these findings while proposing creatinine decline as a simple, low-cost, and widely available parameter that could complement existing risk stratification strategies. Nevertheless, although mortality was higher among patients with slow declines, this association is likely multifactorial and influenced by comorbidities, age, and overall disease burden [34].

Multivariable regression further identified younger age, higher serum albumin, and shorter hospital stay as independent predictors of renal recovery, alongside faster creatinine decline. These results emphasize that not only renal-specific but also systemic factors, including nutritional status, inflammation, and overall clinical stability, play key roles in long-term prognosis [35]. Such findings are consistent with the broader literature, which has repeatedly demonstrated that age and comorbidity burden are central determinants of post-AKI renal recovery [13,36].

Furthermore, longitudinal modeling confirmed that renal function trajectories differed significantly between groups over time. Both time and group effects remained statistically significant, and a meaningful interaction indicated that patients with rapid creatinine decline experienced greater improvement in eGFR throughout the 12-month follow-up period. By accounting for within-subject correlations and missing-at-random data, this approach provided a more accurate representation of renal recovery dynamics. Collectively, these findings reinforce the prognostic role of creatinine decline kinetics and demonstrate that their association with renal recovery persists beyond hospital discharge.

Another aspect deserving attention is the limited availability of novel biomarkers in many regions, especially in developing countries where the prevalence of CKD is rising rapidly. Although biomarkers such as NGAL, KIM-1, and cystatin C can offer earlier detection of renal injury, their high cost and limited accessibility hinder widespread use. In this context, the creatinine decline rate may represent a pragmatic and equitable alternative, leveraging a universally available laboratory test to support timely nephrology follow-up and early initiation of organ-protective therapies.

Furthermore, our results differed slightly from some prior reports that demonstrated stronger associations between slow recovery and mortality. Possible explanations include differences in patient demographics, AKI etiologies, and treatment practices. Our cohort included a higher proportion of sepsis- and surgery-related AKI cases, which may exhibit variable recovery kinetics compared with drug-induced or ischemic etiologies. Additionally, the exclusion of patients on chronic dialysis and the relatively long follow-up period may have enriched our dataset for those with reversible injury.

Future research should integrate additional laboratory variables such as phosphate, potassium, and acid-base balance, which may provide further insight into renal recovery dynamics. Moreover, inclusion of albuminuria (preferably via urine albumin-to-creatinine ratio) would allow a more comprehensive evaluation of CKD risk and disease staging.

The potential clinical implications of our study warrant careful consideration. Monitoring creatinine decline prior to discharge may help clinicians identify patients at higher risk of incomplete recovery who would benefit from closer nephrology follow-up and early interventions. Because this parameter can be derived from routine laboratory tests, it does not incur additional costs and could be easily integrated into clinical practice. However, before creatinine decline can be adopted as a prognostic tool, further validation in prospective, multicenter studies with standardized protocols is required [37].

## 5. Conclusions

In conclusion, our study suggests that the rate of creatinine decline is an independent predictor of renal recovery following AKI and may serve as a practical and cost-effective prognostic indicator. Its simplicity and reliance on routinely available laboratory values make it attractive for clinical application. Nonetheless, these findings should be interpreted with caution, given the retrospective, single-center design and absence of biomarker comparison. Future prospective, multicenter studies with standardized measurement strategies are necessary to validate the prognostic value of creatinine decline and to determine whether it can be integrated into routine clinical decision-making. If confirmed, this parameter could provide clinicians with a valuable adjunct in post-AKI risk stratification and patient management.

## Figures and Tables

**Table 1:** Demographic and clinical characteristics of the study population.

Variable	Total (*n* = 817)	Rapid Decline (*n* = 409)	Slow Decline (*n* = 408)	*p* Value
Age (years)	66.0 ± 13.0	63.9 ± 12.7	68.2 ± 13.0	<0.001
Female sex, *n* (%)	376 (46.0)	168 (41.1)	208 (51.0)	0.002
Hypertension, *n* (%)	474 (58.0)	214 (52.3)	260 (63.7)	<0.001
Diabetes mellitus, *n* (%)	278 (34.0)	120 (29.3)	158 (38.7)	0.003
Cardiovascular disease, *n* (%)	180 (22.0)	78 (19.1)	102 (25.0)	0.030
ICU requirement, *n* (%)	229 (28.0)	92 (22.5)	137 (33.6)	<0.001
Length of hospital stay (days)	9 [6–14]	8 [6–12]	10 [7–15]	<0.001

ICU: intensive care unit. Statistical tests: independent samples *t*-test, Mann–Whitney U-test, and chi-square test as appropriate.

**Table 2:** Etiology of acute kidney injury in the study cohort.

Etiology of AKI	*n* (%)
Sepsis	261 (32.0)
Ischemic injury	221 (27.0)
Nephrotoxic exposure	155 (19.0)
Postoperative causes	98 (12.0)
Multifactorial etiology	82 (10.0)

AKI: acute kidney injury.

**Table 3:** Laboratory parameters and creatinine decline rate.

Parameter	Total (*n* = 817)	Rapid Decline (*n* = 409)	Slow Decline (*n* = 408)	*p* Value
Peak creatinine (mg/dL)	3.1 ± 1.4	3.0 ± 1.3	3.2 ± 1.5	0.070
Discharge creatinine (mg/dL)	1.7 ± 0.8	1.4 ± 0.6	2.0 ± 0.9	<0.001
Creatinine decline rate (mg/dL/day)	0.19 [0.12–0.30]	0.28 [0.22–0.36]	0.12 [0.08–0.15]	<0.001
Urea (mg/dL)	64 ± 24	58 ± 20	70 ± 26	<0.001
Albumin (g/dL)	3.4 ± 0.6	3.6 ± 0.5	3.2 ± 0.6	<0.001
Hemoglobin (g/dL)	10.9 ± 1.8	11.2 ± 1.7	10.6 ± 1.8	<0.001
Sodium (mmol/L)	138 ± 4	138 ± 4	137 ± 5	0.120
Potassium (mmol/L)	4.5 ± 0.6	4.4 ± 0.5	4.5 ± 0.6	0.040

Statistical tests: independent samples *t*-test or Mann–Whitney U-test.

**Table 4:** Post-discharge clinical outcomes.

Outcome	Rapid Decline (*n* = 409)	Slow Decline (*n* = 408)	*p* Value
CKD at 3 months, *n* (%)	98 (24.0)	156 (38.2)	<0.001
CKD at 6 months, *n* (%)	90 (22.0)	148 (36.3)	<0.001
CKD at 12 months, *n* (%)	76 (18.6)	140 (34.3)	<0.001
Mortality at 3 months, *n* (%)	18 (4.4)	32 (7.8)	0.040
Mortality at 6 months, *n* (%)	26 (6.4)	44 (10.8)	0.030
Mortality at 12 months, *n* (%)	38 (9.3)	58 (14.2)	0.030
Readmission within 12 months, *n* (%)	122 (29.8)	168 (41.2)	<0.001
Dialysis within 12 months, *n* (%)	14 (3.4)	36 (8.8)	0.002

CKD: chronic kidney disease. Statistical tests: chi-square test and Fisher’s exact test as appropriate.

**Table 5:** Independent predictors of renal recovery at 12 months (multivariate logistic regression).

Variable	OR	95% CI	*p* Value
Age (per 10 years)	0.78	0.68–0.90	0.001
Female sex	0.86	0.66–1.13	0.280
Hypertension	0.74	0.57–0.96	0.020
Diabetes mellitus	0.71	0.54–0.94	0.015
Albumin (per 1 g/dL increase)	1.42	1.17–1.72	<0.001
Peak creatinine (per 1 mg/dL)	0.94	0.83–1.07	0.330
Creatinine decline rate (per 0.1 mg/dL/day)	1.36	1.22–1.53	<0.001
ICU admission	0.67	0.50–0.90	0.008
Length of hospital stay (per day)	0.98	0.96–0.99	0.010

OR: odds ratio; CI: confidence interval; ICU: intensive care unit.

**Table 6:** Longitudinal analysis of eGFR using the Generalized Estimating Equations (GEE) model.

Parameter	β (Estimate)	SE	Wald χ^2^	*p* Value
Time effect (3–12 months)	0.42	0.14	8.9	0.004
Group effect (rapid vs. slow)	1.28	0.29	19.6	<0.001
Interaction (group × time)	0.37	0.15	5.6	0.018

GEE: Generalized Estimating Equations; eGFR: estimated glomerular filtration rate; SE: standard error. Model type: identity link with exchangeable correlation structure; robust standard errors were used.

## Data Availability

The data presented in this study are available on reasonable request from the corresponding author. The data are not publicly available due to ethical and privacy restrictions.

## Abbreviations

AKI	Acute Kidney Injury
CKD	Chronic Kidney Disease
ESKD	End-Stage Kidney Disease
KDIGO	Kidney Disease: Improving Global Outcomes
eGFR	Estimated Glomerular Filtration Rate
ICU	Intensive Care Unit
CRP	C-Reactive Protein
BMI	Body Mass Index
SD	Standard Deviation
IQR	Interquartile Range
ROC	Receiver Operating Characteristic
OR	Odds Ratio
CI	Confidence Interval
